# Quantitative Field Emission Imaging for Studying the Doping-Dependent Emission Behavior of Silicon Field Emitter Arrays

**DOI:** 10.3390/mi14112008

**Published:** 2023-10-28

**Authors:** Andreas Schels, Florian Herdl, Matthias Hausladen, Dominik Wohlfartsstätter, Simon Edler, Michael Bachmann, Andreas Pahlke, Rupert Schreiner, Walter Hansch

**Affiliations:** 1 Faculty of Electrical Engineering and Information Technology, University of the Bundeswehr Munich, 85577 Neubiberg, Germany; florian.herdl@unibw.de (F.H.);; 2 Faculty of Applied Natural and Cultural Sciences, Ostbayerische Technische Hochschule Regensburg, 93053 Regensburg, Germany; matthias.hausladen@oth-regensburg.de (M.H.);; 3 Ketek GmbH, 81737 Munich, Germany

**Keywords:** field emission, field-emitter arrays, field emission imaging, silicon field-emitter array, doping dependent, p-type silicon

## Abstract

Field emitter arrays (FEAs) are a promising component for novel vacuum micro- and nanoelectronic devices, such as microwave power amplifiers or fast-switching X-ray sources. However, the interrelated mechanisms responsible for FEA degradation and failure are not fully understood. Therefore, we present a measurement method for quantitative observation of individual emission sites during integral operation using a low-cost, commercially available CMOS imaging sensor. The emission and degradation behavior of three differently doped FEAs is investigated in current-regulated operation. The measurements reveal that the limited current of the p-doped emitters leads to an activation of up to 55% of the individual tips in the array, while the activation of the n-type FEA stopped at around 30%. This enhanced activation results in a more continuous and uniform current distribution for the p-type FEA. An analysis of the individual emitter characteristics before and after a constant current measurement provides novel perspectives on degradation behavior. A burn-in process that trims the emitting tips to an integral current-specific ideal field enhancement factor is observed. In this process, blunt tips are sharpened while sharp tips are dulled, resulting in homogenization within the FEA. The methodology is described in detail, making it easily adaptable for other groups to apply in the further development of promising FEAs.

## 1. Introduction

Field emission electron emitters play an important role in vacuum nanoelectronics, due to their high brightness [1,2], coherence [2,3], and narrow energy distribution [2,3,4]. Together with their low power consumption [5,6,7], fast switching frequency [5,6,7], and low thermal load [5,6,7], they can replace thermal emitters in many applications, as well as open up completely new fields. Attractive applications include X-ray devices [8,9], terahertz sources [10,11], or vacuum channel transistors [12,13], among others.

Many of these applications require high emission currents under sometimes poor vacuum conditions. However, high current densities usually lead to short lifetimes and insufficient current stability of the cold cathode due to various degradation mechanisms [14,15,16]. To reduce the current load locally, field emitter arrays (FEAs) consisting of a large number of individual emission sites are often used. Many research groups are focusing on carbon nanotubes as the emitter material, since their high aspect ratio geometry, conductivity, and thermal robustness provide excellent field emission conditions [17,18]. However, it has recently been reported that silicon-based FEAs can achieve similar current densities [19,20,21]. Due to their well-established fabrication methods and integrability into existing technology, silicon-based FEAs are therefore still being studied theoretically and experimentally [13,22].

Regardless of the cathode material, emission uniformity across the array is critical to avoid insufficient FEA lifetime due to high local current densities. Unfortunately, the possibilities to measure emission uniformity are limited. Cathodoluminescent phosphor screens are often used for this purpose [3,14,23,24,25,26,27]. However, limited spatial resolution depending on the grain size [3,26], possible evaporation and burn-out of the luminescent layer [3,28], changes in the saturation behavior of p-doped FEAs due to the luminescence [29], and saturation effects especially over many orders of magnitude of emission currents [30] limit their applicability, particularly for quantified measurements. Fully quantifiable approaches are often based on scanning anode field emission microscopy [26,31,32,33]. Although they offer a high resolution, they cannot provide insight into the actual behavior of the FEA during integral operation and therefore neglect the interaction of active emitters in the array. In addition, these methods use very sophisticated setups, resulting in costly and time-consuming measurements. Commercially available cathode emission profilers, which rely on an anode-hole scanning method, allow for precise measurement of the local emission current during integral operation [34,35]. However, the resolution is limited by the aperture size, which is usually in the tens of microns range, and the scanning mode results in a non-simultaneous analysis of individual emission sites.

To overcome these drawbacks, we recently reported on a low-cost and easy-to-use method for uniformity measurements of FEAs based solely on the commercially available Raspberry Pi HQ camera [36]. It was shown that the use of multiple shutter speeds can extend the dynamic range of the CMOS sensor to allow spatially resolved mapping of emission currents ranging over many orders of magnitude, without sacrificing near real-time and simultaneous observation of the entire array during the integral measurement. The camera can be integrated into almost any standard FEA measurement setup, making it widely accessible to the community. In this paper, we further explore the unprecedented insights provided by this measurement method through a comparison of differently doped FEAs. Comparisons of the characteristics of each individual tip before and after a constant current measurement, as well as tracking the current distribution in the array during the measurement, will provide information on the degradation mechanisms of the FEAs.

## 2. Materials and Methods

**Figure 1:**
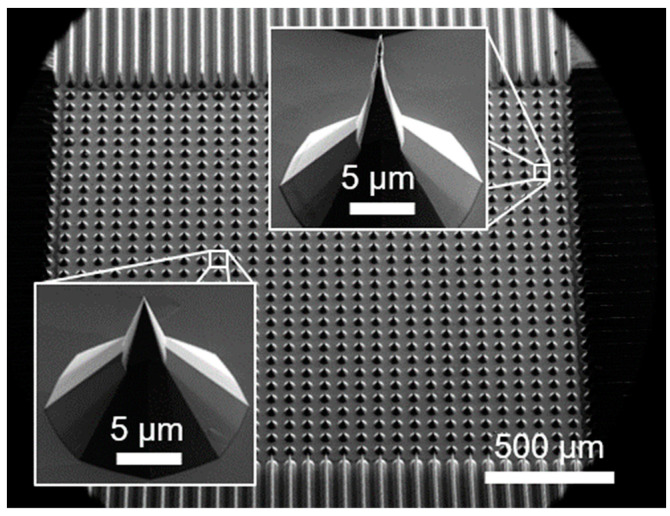
SEM image of one of the three 30 × 30-tip FEAs measured in this study. The insets show two selected tips magnified to highlight the rare but possible geometric differences between the individual tips in one array.

The investigated FEAs were fabricated by wafer dicing and wet chemical TMAH etching [37] of n (phosphorus, resistivity of 10–20 Ωcm), n^++^ (antimony, resistivity of <0.005 Ωcm) and p (boron, resistivity of 10–20 Ωcm) doped Si <100> substrates. Fabrication parameters were adjusted to produce morphologically comparable arrays of 30 × 30 pyramidal-shaped tips for each doping with an average height of approximately 30 µm and a pitch of 66.7 µm. The 900-tip array covers a total area of 2 mm × 2 mm on a 4 mm × 6 mm die. Figure 1 shows an overview SEM image of such an array. As can be seen from the insets, the geometric differences within an array are rare but can be quite significant due to the fabrication.

The measurements are performed in a vacuum setup with a controlled chamber pressure of 10^−5^ mbar. This relatively high pressure for field emission measurements was chosen to investigate the emission behavior of the FEAs under real application conditions [37]. The main component of the measurement setup is the Raspberry Pi HQ camera (Raspberry Pi Ltd., Cambridge, UK), which is equipped with the IMX477 back-illuminated CMOS imaging sensor [38] with 4056 × 3040 pixels and a pixel diameter of 1.55 µm. Compared to the size and pitch of the tips, the sensor therefore provides sufficient resolution to measure the electron emission of the FEAs. To ensure sufficient signal transmission, the surface of the sensor must be exposed by removing the camera body, the lens, and the infrared filter. A diode setup is used with the camera serving as the extraction anode. To do this, the FEA is placed directly on the 6.287 mm × 4.712 mm sensor surface, together with a 60 µm thick mica sheet sandwiched in between for electrical insulation. The potential of the camera is set to ground. The FEA is pulled to a negative potential by a regulation circuit [36,39] which allows current-regulated measurements, but limits the extraction voltage to a maximum potential of −1 kV.

There are three parts to the herein-used measurement procedure. Initially, a current–voltage (IV) characteristic is obtained by conducting seven successive current-controlled IV sweeps (four up sweeps, three down sweeps) ranging from 5 nA to 1 µA. This is followed by a constant current measurement (CCM) at 1 µA for one hour. The duration and current level of the CCM were chosen arbitrarily with the intention of causing comparable degradation in the FEAs studied, but still ensuring their survival during the measurement if possible. If a measured FEA fails to achieve the control current for ten consecutive measurement points within the available extraction voltage of 1 kV the CCM is programmed to terminate. Finally, another IV characteristic is recorded to analyze potential changes in the emission behavior of the FEA resulting from the CCM. Each individual sweep consists of 40 measurement points, with each electrical data point recorded twice to account for fluctuations in the FEA and measurement setup during the four to six seconds needed for image acquisition. In addition to the current measurement, a high-impedance voltage divider is used to measure the required extraction voltage applied to the FEA. In the post-processing, all related measurement points are averaged and given an uncertainty by the empirical standard deviation.

In parallel with the electrical measurement, the camera takes three raw images at each data point for four different exposure times (31.5 ms, 10 ms, 3.15 ms, and 1 ms) each, giving a total of 12 images per measurement point. As noted above, this process takes approximately four to six seconds, mostly due to the time needed for the camera to adjust the exposure time. To eliminate possible time-related fluctuations in individual tip emissions during this time, the three snapshots taken at different times during acquisition for each shutter speed are later combined by averaging during post-processing. To achieve faster acquisition times, reducing the number of frames and shutter speeds can be considered. The range of exposure times has been empirically determined to efficiently measure the typical single-tip currents of this FEA in the range of tens of picoamperes to several hundred nanoamperes. Note that the optimal shutter speeds may vary for other FEAs and control currents. To minimize the data size and processing time for image storage, the resolution is artificially reduced for each image captured. This is done by combining each 4 × 4 of the original 12-bit pixels into one 16-bit pixel. In addition, the image section is cropped by 50% as the active emission area of the FEAs only covers the inner area of the sensor.

During post-processing, the images are fed through a spot detection algorithm [36] that also analyzes the gray values of the spots generated by the electron emission for saturated pixels. In the case of pixel saturation, the entire spot is replaced by extrapolating the data captured at a lower exposure time. This is based on the linear sensor response of the camera for constant input currents at different shutter speeds, which was proven earlier [40]. The corrected images are then used to determine the corresponding currents of each spot. This is done by multiplying the integral gray value of each spot by the quotient of integral current and integral brightness over the entire image. This gives a distribution of the individual tip emission currents over the entire array for each data point [36,40].

In order to reconstruct the characteristics of each of the individual tips (see Section 3.3), another algorithm uses the spatial resolution of the sensor. Starting with the first measurement point, the algorithm stores the center of each detected spot in the image as a list of found tip positions. The currents calculated from the spot pixel gray values are then assigned to the respective spot positions. For all subsequent measurement points, the occurring spots are compared with the tip positions already listed. If a spot lies within a radius of 6 pixels around the center of a listed tip in the reduced image (24 pixels in the original image), the spot current is assigned to this tip. Otherwise, a new tip position is added to the list, together with the corresponding current value. The radius of 6 pixels was chosen because it corresponds to a physical distance of 37.2 µm, which is approximately half the distance between two tips in the FEA.

By using Murphy–Good coordinates, the field enhancement factor (FEF) of each actively emitting tip is determined from the slope of a linear regression. This was carried out according to the method proposed by Forbes [41]. The electron affinity of silicon (4.05 eV) was used as the work function to match the semiconducting properties of the substrate material. As field emission is subject to fluctuations, not all datasets of each individual tip are suitable for linear regression. Therefore, only curves with more than ten measurement points are taken into consideration. Additionally, if the calculated error of the extracted FEFs is greater than 500 or exceeds the FEF value itself, they will be disregarded.

## 3. Results and Discussion

### 3.1. Integral Measurements and Single-Tip Emissions

**Figure 2:**
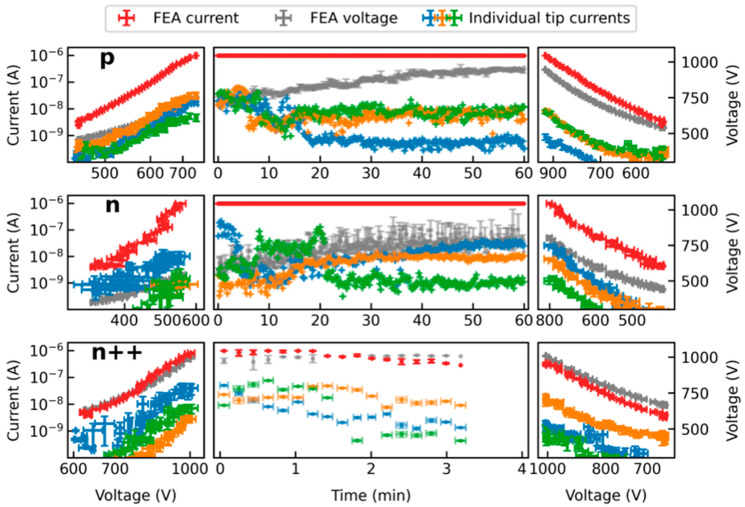
Measurement results for three differently doped but morphologically similar FEAs including the emission currents of three selected individual tips of each FEA obtained from the optical data. The characteristic IV curves before and after the CCM on the left and right, respectively, are plotted in Millikan–Lauritsen coordinates with reciprocal distances instead of reciprocal voltages for better readability.

Figure 2 shows the result of the measurement for three differently doped but geometrically similar FEAs. The left and right part of this illustration represents the characteristic IV curve before and after the CCM measurement, respectively. The middle part shows the applied voltage and the current during the CCM. The sweeps before and after CCM (left and right) are plotted in Millikan–Lauritsen coordinates [42], where the *x*-axis has been relabeled and rescaled with reciprocal distances to show the voltage instead of its inverse. Furthermore, the *x*-axis of the left part is mirrored. In addition to the integral current (red) and the integral voltage (gray), Figure 2 also shows the characteristics of three individual tips for each FEA (blue, orange, and green). These tips were selected to provide a comprehensive representation of the diverse range of current courses at individual emission sites. The currents are calculated from the image data captured by the camera, as described above.

It is important to note that the calculation of the currents from optical data relies on a simplified approach. Even if the extraction voltage is assumed to be constant during the image acquisition time, local differences may occur in the array, e.g., due to a potential voltage drop across the space charge region during saturated emission [43]. This results in differences in electron energy affecting sensor brightness and causing distortion in the calculated currents. Furthermore, there may be charging effects from non-conductive layers, changes in transmission rates resulting from deposition or ablation from the sensor surface during measurement, and potential variations in pixel sensitivity. Consequently, inaccuracies might occur in the optical data, particularly at high current densities. However, the measurements presented in this study were conducted at low integral currents, and the reconstructed individual tip currents do not show any unusual behavior. Nevertheless, these currents should be interpreted in terms of their magnitude instead of their absolute value until further research is conducted.

The measurement of the p-doped emitter is presented in the top row of Figure 2. Prior to the CCM, saturation behavior can be observed. This is due to the fact that p-type and low n-type doping can limit the maximum emission current at high electric fields due to the limited number of charge carriers in the conduction band [29,43,44]. The level of the current saturation hereby strongly depends on the charge carrier generation in the depletion region at the surface, which can be influenced e.g., by thermal or photonic excitation [29,45]. In the case of this measurement, the expected strongly pronounced adsorbate coverage of the tips due to the high pressure in the vacuum chamber can also have a large effect on this value [43]. Additionally, it has been shown that geometric differences can influence current saturation, which might also be the case here [29]. For the herein-measured p-doped FEA, the saturation level can be mostly observed in the range of 1 nA to 10 nA. For the reasons mentioned above, saturation can also be significantly higher at times (see orange and blue curves). However, at the beginning of the CCM, the FEA temperature increases due to the constant current load, which in turn favors the desorption of adsorbates. In the case of the orange and green tips, this leads to the recovery of the stable emission level in the range of 10 nA. In some cases, however, the excessive emission current also leads to irreversible degradation, as can be seen from the tip plotted in blue.

A similar behavior can be observed for the n-doped FEA. Emission currents at or below 10 nA usually do not cause significant degradation to the tip. Overly high emission currents result in either severe degradation (green tip) or burn-in to a constant emission level (blue tip). However, the orange graph shows that the progressive current load during the CCM does not necessarily lead to a deterioration of the individual tips. In fact, for all FEAs, many tips can be identified that improve over the course of the measurement and, in some cases, despite being activated later, can still contribute a relevant proportion of the total current. This will be further discussed in detail in Section 3.3.

Apart from this, it is noticeable from Figure 2 that the highly doped n^++^-type FEA only reached the control current for about 3 min during the CCM, while the p-type and the lowly doped n-type FEA were able to emit for the whole hour at 1 µA and 10^−5^ mbar within the available extraction voltage range. The highly n-doped FEA barely reached the integral control current during the sweeps and started the CCM with an extraction voltage of 1000 V already. On the one hand, this may be due to random geometric differences, since, for example, a lower tip height, due to the slightly different etch times in the fabrication of the FEAs may have a negative impact on both the macroscopic field and the FEF. On the other hand, too much degradation during the initial sweeps may also be the reason. This will be discussed in more detail in Section 3.2.

**Figure 3:**
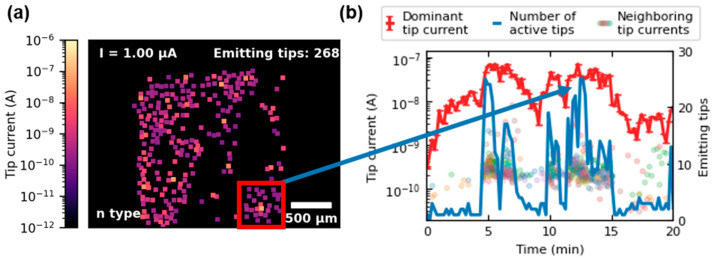
Clustering phenomenon illustrated for the n-type FEA during the CCM. In (**a**), the emission heat map of minute 13 of the CCM is shown. The red box marks the area where the clustering is observed. The graph in (**b**) plots the current of the dominant tip (red) over the first 20 min of CCM together with the number of emitting tips (blue) inside of the box. The emission currents of the less dominant tips in the box are indicated as multicolored dots.

An intriguing phenomenon has been increasingly observed in the case of n^++^- and n-doped FEAs. By analyzing image data over the course of the CCM, it is apparent that strong emitting tips often activate the emission of their surrounding tips. This effect can be illustrated with Figure 3a, which displays the emission heat map from minute 13 of the CCM of the n-type FEA. The markers’ color reflects the emitted current of each active tip of the array. Their size corresponds to the area that is integrated when reading the distinct emission spot’s gray values. The area where clustering occurs is marked with a red box. In Figure 3b, the red line represents the current of the dominant tip (identified as the yellow emission spot in the heatmap) over the first 20 min of the CCM. The blue line indicates the number of actively emitting tips within the box during this time. The currents of other tips in the region are displayed as dots in multiple colors. A correlation between the current increase in the dominant tip to the number of active tips within the box can be observed. Similar behavior has previously been documented in the literature, observed during discharge analysis of CNT field emission cathodes [14]. This phenomenon could be attributed to the rising ionization of residual gas molecules caused by the increasing emission of the dominant tip. As a result, these ions may stimulate nearby tips to emit by raising the local potential through space charge. Additionally, higher rates of desorption resulting from ion bombardment or local temperature increases near the dominant tip because of Joule heating may also serve as explanations. This effect spreads radially, and emission from neighboring tips fluctuates considerably. However, they rarely contribute more than a few thousandths to the integral emission current, while the dominant tip carries up to 6%. Clustering was mainly observed in n^++^- and n-doped emitters, while it occurred less frequently in p-type FEAs due to the uniformity induced by saturation, which prevents individual tips from dominating.

### 3.2. Change of Current Distribution during the Measurement

**Figure 4:**
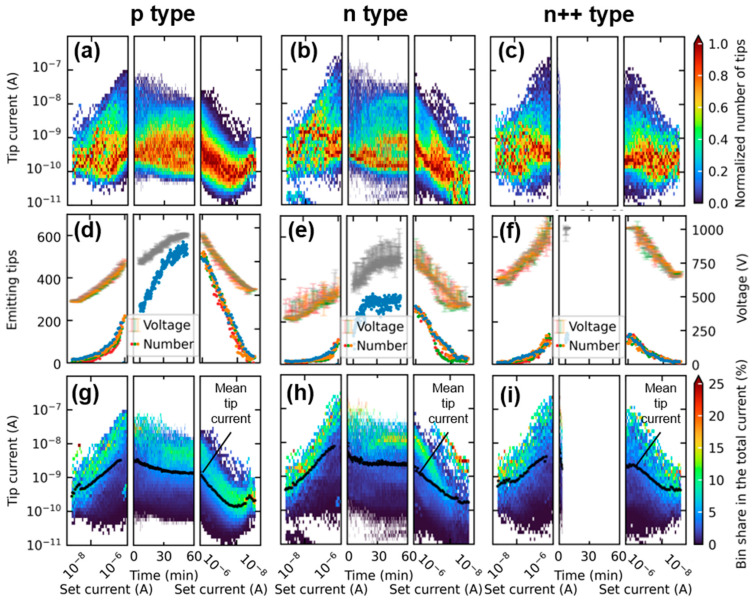
(**a**–**c**) illustrates the normalized current distribution of the three measured FEAs for every measurement point before, during, and after the CCM as a pseudocolor plot. In (**d**–**f**), the number of actively emitting tips is plotted together with the respective extraction voltage. (**g**–**i**) represents the center of the distribution in terms of each group’s share of the FEA emission current, displaying the accumulated percentage of all the tips in each bin to the integral current.

The measurement method yields a current distribution for each data point, which can be evaluated for example spatially as a heat map or statistically as a histogram with categorization based on the emitted current per tip [36,40]. In order to compare the changes in current distribution over the course of the sweeps and the CCM, a uniform bin size must be set. A viable approach is to distribute ten bins per magnitude linearly on a logarithmic scale. To illustrate the evolution of the resulting histograms for each data point throughout the measurement cycle, a pseudocolor plot is used as shown in Figure 4a–c. In these plots, each pixel’s height is determined by the pre-established bin boundaries from previous histograms, and the width corresponds to the distance between successive data points. Unlike in Figure 2, the *x*-axis during the current-controlled sweeps (left and right) represents the respective FEA set current. Since these characteristics consist of seven partial sweeps, each data point distribution contains seven sub-distributions that are added together. For comparison to the CCM, each data point distribution is then normalized to the respective maximum number of tips per bin. Consequently, the color shows the numerical center of each distribution and displays the group of tips represented in the greatest number in a comparable way for the entire measurement, including CCM. Figure 4d–f displays the number of tips emitting simultaneously at each data point along with the extraction voltage necessary to attain the control current. This number is graphed for each sweep in a different color during the characteristics before and after the CCM. Figure 4g–i depict the percentage of each bin’s contribution to the total integral current at each measurement point. To accomplish this, the cumulative current of all individual tips within each bin was divided by the total current per distribution. Furthermore, the average tip current of the entire FEA is represented in black.

The overall shape of the current distributions in Figure 4a–c is comparable among all three dopants. During the initial characteristics, the distributions show a high degree of scattering, but stabilize during the CCM. In the subsequent characteristics, the distributions are narrower with less prominent maximum currents. Part of this stabilization is attributable to various conditioning effects [45,46] affecting the emission of the individual tips during the initial operation. Additionally, from Figure 4d–f, it is apparent that the number of emitting tips increases significantly over the course of the measurement, due to the increasing voltage. Analyzing only the sweeps before the CCM, we observe a rapid and reproducible activation of up to 250 tips for the p-doped FEA with comparable extraction voltages for each sweep. In contrast, for the n^++^ and n FEA, only a limited number of tips contribute to the emission current simultaneously across a wide set current and extraction voltage range, reaching a maximum of about 125 tips. Furthermore, the voltage data points in Figure 4e,f regarding n^++^- and n-type are more widely scattered, indicating more fluctuation and degradation effects between the individual sweeps of the characteristic.

Notable differences in the number of simultaneously emitting tips during the CCM are observed in Figure 4d,e. The p-type FEA continually activates more tips, while the n-type FEA reaches its maximum at approximately 300 individual emitters after around 20 min. At about the same time, distinct levels of strongly represented emitter groups start to form in the distribution course shown in Figure 4b. From this point on, further increase of the extraction voltage induces many of the already active tips to emit higher currents and/or counters their degradation in terms of emission current. As a result, an increase in the number of emitters producing more than 10 nA can be observed in the CCM part of Figure 4b, in addition to the numerous tips emitting between 100 pA to 1 nA. Unlike this, the number of tips emitting simultaneously in the p-type FEA increases continuously and reaches over 500 by the end of the CCM. This corresponds to over 55% of the tips in the array. According to Figure 4a, the increased number of tips results in a more continuous distribution of emitted currents. However, it is worth noting that the tips emitting currents within the range of 100 pA to 1 nA are most strongly represented here as well.

Another significant difference between the dopings can be observed in the maximum currents in Figure 4a–c, reaching values of over 200 nA per tip for the n- and n^++^-type FEA and only up to 100 nA for the p-type tips during the initial sweeps. However, it cannot be excluded that this difference is due to a possible voltage drop over the space charge region of p-doped emitters in saturation. During CCM, the maximum currents decrease significantly for both the n- and p-type FEA. Note that the n^++^-type FEA is excluded from the comparison because its CCM ended after two minutes due to the extraction voltage limit. Apart from the expected high degradation of the strongly emitting tips regardless of doping, both FEAs depicted in Figure 4a,b may display similar behavior due to the potential combination of adsorbates and thermal runaway. The measurements took place at a high chamber pressure of 10^−5^ mbar, which may have caused the accumulation of numerous adsorbates on the surface of the emitting tips during the initial sweeps. The presence of adsorbates away from the tip apex affects the generation of charge carriers on the surface, leading to a possible increase in the current limit of p-type FEAs [43]. Moreover, the presence of adsorbates at the tip apex can lead to a decrease in current due to a change in the work function [46]. As the constant current heats the tip during the initial minutes of CCM, the desorption rate rises. At the same time, the elevated temperature results in a rising generation of charge carriers. Depending on the surface conditions, temperature, and doping, the typical current saturation for p-type FEAs, which is in the range of a few nanoamperes, can be restored (refer to Figure 2, p-type, orange graph), or the tip current continues to increase until it degrades because of thermal runaway (refer to Figure 2, p-type, blue graph). For the n-type FEA, various types of degradation can be observed, as demonstrated by its individual tip characteristics presented in Figure 2. A more comprehensive analysis of these degradation types will be discussed in Section 3.3.

The figures displayed in Figure 4g–i demonstrate the respective contributions made by each group to the total FEA current. It is well known in the literature that most of the FEA current is typically carried by a small number of very sharp tips, especially at low voltages [47,48,49]. Particularly during the characteristics before the CCM, this is true for all the examined FEAs, independent of the doping. During CCM, however, differences between the p- and n-type emerge. Especially at the beginning, the n-type FEA relies mainly on its tips emitting around 100 nA. However, as the measurement continues and the extraction voltage increases, many of those tips degrade due to their high current load. After approximately 20 min of CCM, a state of equilibrium is reached in which the distributions are centered around the tips emitting slightly more than 10 nA. This phenomenon is less evident for the p-type FEA, as current saturation suppresses excessive emissions from the beginning, forcing the emitter to spread the current load over more tips. This helps to prevent premature burn-out and associated degradation. The equilibrium state, which is achieved after around 30 min, aligns well with the expected current saturation levels typically around 1 nA to 10 nA, as previously stated. However, activating the additional tips requires a higher electric field, causing the p-doped emitter to exhibit a faster increase in extraction voltage than the n-doped one.

Upon closer analysis of the data, it is evident that at the start of the CCM, only 20% of the current for p-type FEAs is carried by tips emitting 10 nA or less. However, as the more highly emitting tips degrade over time, this percentage steadily increases to 90% by minute 30, where it remains until the end of the measurement. Similarly, at the beginning of the CCM, 20% of the set current for the n-type FEA comes from tips emitting 10 nA or less. However, the value increases linearly to only 50% until minute 20 and then fluctuates around this average value. The remaining current share for the n-type emitter of 80% at the beginning of the CCM and 50% at the end is distributed over only 15 and 20 tips, respectively. For the p emitter, however, this value drops from 25 tips carrying 80% initially, to only 5 tips carrying the residual 10% of the integral current by emitting over 10 nA in the end. This study confirms that, firstly, the saturation effects of p-doped emitters can notably enhance the uniformity of FEAs. Secondly, the enhanced uniformity also results in steady emission and prolonged lifespan of FEAs through the spread of current load across numerous tips.

### 3.3. Individual Tip IV Characteristics

**Figure 5:**
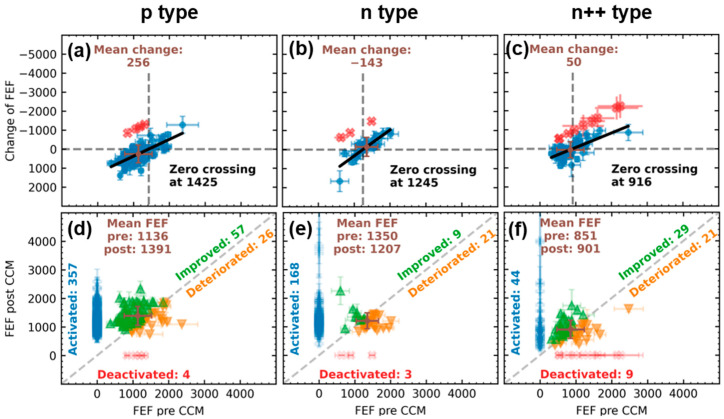
Comparative study of the change in single-tip FEFs. In (**a**–**c**) the difference between the FEF pre- and post-CCM is plotted versus the initial FEF. (**d**–**f**) show the FEF post-CCM versus the FEF pre-CCM, together with the newly activated and during the CCM deactivated tips. Note that the CCM of the n^++^-type FEA only lasted for three minutes due to the limited extraction voltage, while the p- and n-type were operated for the full 60 min at 1 µA.

For a more comprehensive understanding of the degradation of individual tips under constant current load during the one-hour CCM, this section will analyze the IV characteristics of the individual tips before and after CCM in greater detail. One parameter that offers itself for that is the FEF. It describes the ratio of the effective field at the apex of the tip to the macroscopically applied field and has been shown to be strongly dependent on the radius of curvature and the height of the emitter [50,51,52]. However, it is also influenced by adsorbates on the surface [46]. Consequently, analysis of changes in the FEF provides insight into the deterioration of individual tips within the FEAs with respect to the geometry and surface properties of the emitter. The result of the comparative study between the three differently doped emitters is shown in Figure 5. In (a–c) the change in the FEF of a tip as a function of its FEF before CCM is depicted. Tips that did not emit after CCM are shown in red. The data points of tips that were active both times (blue) were fitted linearly, in order to determine their zero crossing. Additionally, their mean change was determined and plotted in brown. Figure 5d–f illustrates in detail how each of these tips has changed individually, as the FEF after the CCM is plotted against the FEF before. In case of an increase (points above the half line), the respective tip is shown in green, and in case of a deterioration (below the half line) in orange. The mean FEF before and after is plotted in brown. In addition, at x = 0, the newly activated tips during the CCM are displayed in blue, and at y = 0, those deactivated during the CCM in red. Again, it should be noted that the values presented should be interpreted relative to each other rather than in absolute terms due to potential unknown uncertainties that may arise in the linear regression of the individual tip characteristics.

From Figure 5a–c, a clear linear relationship between the initial FEF and degradation (change in FEF) can be observed, independent of doping. Higher FEFs typically correspond to sharper tips and higher electric fields at the apex [50,51,52]. Therefore, these emitters tend to emit earlier and more current, resulting as expected in higher degradation. Noteworthy is the zero crossing, which corresponds to the threshold above which a tip is more likely to degrade than to improve. For the n^++^-type FEA, this threshold FEF is 916, for the n-type it is 1245, and for the p-type it is 1425. This indicates that the current limitation of p-doped emitters might prevent higher FEF tips from degradation. Furthermore, the average change of active tips before and after CCM (plotted in brown) is also positive for the p-type emitter, which indicates an improvement in the average, while the n-type emitter degrades on average. The average change for the n^++^-type FEA is also slightly positive. However, when interpreting the n^++^-type data, it is essential to note that this FEA was only subjected to three minutes of CCM. Therefore, the degradation cannot be compared to the other two FEAs, as the total current load is different.

A comparison of Figure 5d–f reveals that the ratio of improving to degrading tips is significantly higher for the p-doped FEA compared to the two n-doped FEAs. Moreover, the activation of 357 new tips outweighs the omission of only four tips in this case. For the n-type FEA, more of the pre-CCM active emitters degrade than improve. However, only three tips are deactivated while 168 new tips are activated. As for the n^++^-doped FEA, the degradation to improvement and activation to deactivation ratios are closer to one, indicating significant fluctuations during the two-minute CCM.

A notable observation is made by combining the two plots. It appears that the mean FEFs after CCM of the constantly active tips almost perfectly approach the respective threshold FEFs from the zero crossing of Figure 5a–c in all three cases. Together with the fact that the number of entirely deactivated tips is almost negligible, it demonstrates that the change of the FEA’s emission in current-controlled operation is not only dominated by a gradual deterioration of the sharpest tips in the array. It rather exhibits a burn-in process towards a particular ideal FEF by both improvement and deterioration of the individual tips. Preliminary measurement results obtained at varying integral current levels during the CCM demonstrate a clear dependence of the threshold FEF on the integral current load. However, a detailed study of this phenomenon is beyond the scope of this work, and reference is made to future work.

## 4. Conclusions

In this study, we demonstrated the capabilities of a novel, easy-to-adapt measurement method for characterizing field emitter arrays (FEAs). It offers the possibility to quantitatively analyze the emission current for individual emission sites during integral operation of the FEA. Since it is based on a commercially available CMOS imaging sensor, it can be integrated into almost every standard vacuum measurement setup. The presented measurement form represents only the most basic configuration and can be enhanced in numerous ways for future research. Three differently doped (p-type with resistivity of 10–20 Ωcm, n-type with resistivity of 10–20 Ωcm and n^++^-type with resistivity of <0.005 Ωcm) but morphologically similar silicon FEAs were investigated using a tripartite measurement, consisting of current-controlled current–voltage (IV) characteristics measurement prior and subsequent to a one-hour constant current measurement (CCM) at 1 µA at a chamber pressure of 10^−5^ mbar. The n^++^-type FEA could only emit the set current for a limited time of about three minutes due to the extraction voltage limit of the setup, which made the outcomes partially incomparable. The analysis of the individual tip emissions revealed a clearly observable saturation behavior of several individual p-type tips, which emitted mostly in the range of 1 nA to 10 nA. This led to a faster and stronger activation of the array and therefore a more stable and uniform current distribution for this kind of doping. During the measurements, a clustering phenomenon was observed in which highly emitting tips led to an increased activation of the surrounding emitters. This effect is more pronounced for the n- and n^++^-type FEA, due to their inferior emission uniformity. The analysis of the distributions revealed that, in accordance with the literature, the integral emission current is heavily dominated by the strongest emitting tips, especially during the initial characteristics for the n- and n^++^-type FEA. Continued conditioning during the CCM led to an equilibrium state being reached for both the p-type and n-type. Here, the majority of the total FEA current was carried by a distinct group of tips emitting over 10 nA for the n-type, while for the p-type, the emission centered around slightly lower current levels that were less discrete. A burn-in process was identified as the cause of this equilibrium state by analyzing the individual tip field enhancement factors obtained from the IV characteristics before and after the CCM. It was demonstrated that both the improvement and deterioration of individual tips result in the mean field enhancement factor approaching a current-dependent optimum value.

## Data Availability

Data is contained within the article in the images and plots. The source code for the control of the measuring station, as well as for the evaluation of the data can be provided by the corresponding author upon reasonable request.
